# Results of contralateral Athens and decentered individualized sphero-cylindrical (DISC) protocols for keratoconus management

**DOI:** 10.3205/oc000261

**Published:** 2025-11-25

**Authors:** Igor Knezović, Nina Jovanović, Sara Djurić

**Affiliations:** 1 Knezović Eye Institute, Zagreb, Croatia

**Keywords:** DISC protocol, Athens protocol, keratoconus, PRK, corneal cross-linking, topo-guided

## Abstract

**Purpose:**

To present the results of two different surgical procedures, decentered individualized sphero-cylindrical protocol (DISC protocol) and Athens protocol, performed on a 34-year-old patient after being diagnosed with keratoconus in both eyes.

**Observation:**

The patient’s left eye was subjected to the Athens protocol (phototherapeutic keratectomy (PTK) + partial topo-guided photorefractive keratectomy (TG-PRK) + corneal cross-linking (CXL)). In contrast, the patient’s right eye was subjected to the DISC protocol (PTK + DISC excimer ablation + CXL). After administration of topical anesthetic, the patient was subjected to PTK of the central 7 mm optical zone with 50 µm depth of epithelial ablation. After excimer laser ablation in both eyes, riboflavin 0.1% solution was applied topically every 2 minutes for 20 minutes. This was followed by five cycles of 5-minute-long irradiation (for 25 minutes) using a UVA 370 nm at 3.0 mW/cm^2^ CXL (CSO VEGA CMB X Linker, Florence, Italy) application. 36 months postoperative uncorrected visual acuity (UCVA) of the left eye remained the same as preoperatively, while the best corrected visual acuity (BCVA) was –3.5 Dsph=20/30. In the right eye, UCVA and BCVA were both 20/22. Results of Fourier’s analysis of the right eye imply a 52% decrease in corneal irregularity, while in the left eye, corneal irregularity decreased by 12.8%. Values of spheric aberration in Zernike analysis 36 months postoperatively showed a lower increase in Athens protocol than DISC protocol. The index of vertical asymmetry and the index of surface variance showed lower values in both procedures during the whole postoperative period, while the index of height decentration showed a more significant decline in Athens protocol 36 months postoperatively.

**Conclusion and importance:**

DISC protocol is a novel approach, with the potential to become a promising strategy for slowing the progression of keratoconus and recovering uncorrected visual abilities.

## Introduction

Keratoconus (KC) is characterized by corneal thinning and bulging, typically beginning in adolescence, and evolving slowly, affecting approximately 86 in 100,000 people [1]. The first-line therapeutic option is refractive correction with glasses or soft contact lenses. With progression, a rigid gas-permeable, scleral lenses [2], or a corneal transplant may be needed [3]. The etiopathogenesis is not well understood, but there are several risk factors linked to the condition, i.e., genetic predisposition, age, Down syndrome, Leber congenital amaurosis, and paternal consanguinity [4]. Strong correlations have been found with trauma occurring with persistent eye rubbing [5]. There are several approaches to treating KC. Kankariya et al. proposed an algorithm of KC treatment that directs treatment based on poor or good visual acuity (VA), directing treatment towards CXL plus (additional protocol next to CXL) in case of poor VA [6]. 

The Athens protocol, validated and reported in the literature with promising long-term results, showed optimal practical application, aiming to normalize the corneal surface, correcting the refractive error and irregular astigmatism [7]. Three crucial steps are included in the Athens protocol. The epithelium is removed, and the corneal surface is regularized during the phototherapeutic keratectomy (PTK). Further, the excimer laser is used to correct the micro-irregularities on the corneal surface (partial topo-guided photorefractive keratectomy (TG-PRK)). The last step is the corneal cross-linking (CXL) technique to stop the progression of keratoconus by increasing the covalent connections between the collagen molecules responsible for mechanical stability [8].

We previously introduced and reported a new method in KC treatment [9], decentered individualized sphero-cylindrical (DISC) protocol designed by the first, senior author (IK), developed and tested at the Knezovic Eye Institute, Zagreb, Croatia. The DISC protocol consists of two laser procedures and individually adapted CXL for each patient. This report aims to compare therapeutic success in one patient treated with DISC and Athens methods in different eyes. DISC method is based on the decentred excimer laser ablations of the patient’s partial sphere-cylindrical refraction from the pupillary center and corneal vertex toward the place of the greatest curvature of the cornea (corneal apex). The exact position of the decentration as well as the magnitude of the laser ablation is defined for each patient individually. The concept behind the decentration were clinical observations during ophthalmological examinations where authors noticed patient’s head moving to obtain better vision. In this case report, the patient was trying to adjust the central part of the trial lens toward the corneal apex instead of the pupil center. In doing so, the patient’s BCVA was improved. Thus, the authors hypothesized that if the location of the laser ablation were shifted in the desired direction, improvement in visual acuity can be expected. 

## Case description

**Figure 1:**
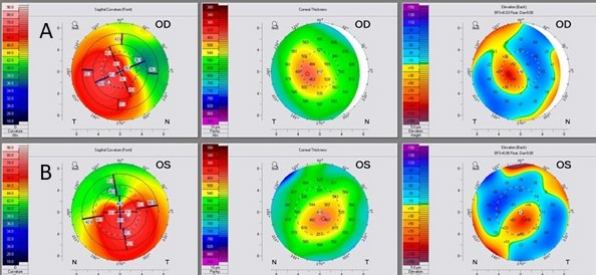
Preoperative Oculyzer images of the right and left eye. A: right eye, B: left eye (Dodati pahimetriju oba oka)

Thirty-four years old male, Caucasian patient was diagnosed with keratoconus at Knezovic Eye Institute, Zagreb, Croatia, with no comorbidities. His bilateral vision disturbances decreased during the previous ten years. The preoperative uncorrected visual acuity (UCVA) of the right eye (OD) and the left eye (OS) was 20/100 both, and the best corrected visual acuity (BCVA) was 20/63 (plan/–2,5 Dcyl ax 67), and 20/63 (–0,5 Dsph/–2,0 Dcyl ax 125), respectively. The patient tried to wear rigid gas permeable (RGP) contact lenses; however, the level of discomfort was high, thus he opted to discontinue the usage. The KC diagnosis was confirmed using corneal tomography imaging (WaveLight^®^ OculyzerTM II, Alcon Surgical, Ft. Worth, Texas, USA). The preoperative patient’s anterior corneal surface curvature, pachymetry, and posterior elevation maps for both eyes are shown in Figure 1 (Fig. 1).

PTK, DISC ablation, and partial TG-PRK were carried out using the Alcon/WaveLight Allegretto Eye-Q 400Hz Excimer Laser platform (Alcon Laboratories, Ft. Worth, Texas, USA). The preoperative protocol included serial topography maps and an algorithm analysis of data. The algorithm involved changing the depth of treatment, modifying the optical zones, and adding a refractive correction that is positioned directly above the line of sight.

### Left eye – Athens protocol

The patient’s left eye was subjected to the Athens protocol (PTK + Partial TG-PRK + CXL) on 25^th^ April 2019. PTK laser ablation of the central 7 mm optical zone and 50 µm depth of epithelial ablation was done after receiving 0.4% oxybuprocaine hydrochloride eye drops as a topical anesthetic. Partial TG-PRK was performed with target refraction of –1.77 Dsph/–2.81 Dcyl ax 116, optical zone 5.0 mm, and transition zone 6.25 mm. 

The maximum ablation depth was 58 µm and the central ablation was 28 µm. Residual corneal thickness was estimated at 455 microns (405 microns + 50 microns epithelium). The stromal surface was treated with 0.02% mitomycin C (MMC) for 40 seconds to prevent the potential corneal haze development. Then, riboflavin 0.1% solution was applied topically every 2 minutes for 20 minutes. This was followed by five cycles of 5-minute-long irradiation (for a total of 25 minutes) using a UVA 370 nm at 3.0 mW/cm^2^ CXL (CSO VEGA CMB X Linker, Florence, Italy) application.

One month after the surgery, the patient’s UCVA was 20/100, and the BCVA (–3,00 Dsph/–1,00 Dcyl ax 80) was 20/63. Three months postoperatively UCVA was20/200 and BCVA (–4,5 Dsph /–1,25 Dcyl ax 100) 20/32. Six months postoperatively UCVA was 20/200 and BCVA (–3,5/–0,5 Dcyl ax 100) 20/32 and 36 months postoperatively UCVA was 20/100 and BCVA (–3.5 Dsph) 20/30 (Table 1 (Tab. 1)). 

### Right eye – DISC protocol

The patient’s right eye was subjected to the DISC protocol (PTK + DISC ablation + CXL) on 3^rd^ October 2019. The procedure started with phototherapeutic keratectomy (PTK) laser ablation of the central 7.0 mm optical zone with 50 µm depth of epithelial ablation after receiving 0.4% oxybuprocaine hydrochloride eye drops as a topical anesthetic. DISC ablation was performed, aiming for a decentration of 200 µm inferior and 200 µm temporal, or roughly 21.86% of the abnormal corneal apex distance. The corneal apex was located 770 µm inferior and 1,040 µm temporal from the pupil center. The magnitude of laser ablation was –1.25 Dsph which is a spherical equivalent (SE) of the patient’s preoperative subjective refraction (plan/–2.5 Dcyl ax 67). The optical zone was 5.5 mm, the transition zone was 6.1 mm, and both maximal and central depth were 13.86 µm.

The residual corneal thickness after the procedure was estimated to be 439 µm, including epithelium. Immediately after the excimer laser procedure, the stromal surface was treated with 0.02% MMC for 40 seconds to prevent the development of corneal haze. Excimer laser ablation was performed within the width of the pupil in mesopic light conditions, followed by riboflavin 0.1% solution application topically every 2 minutes for 20 minutes. This was followed by five cycles of 5-minute-long irradiation (for a total of 25 minutes) using a UVA 370 nm at 3.0 mW/cm^2^ CXL (CSO VEGA CMB X Linker, Florence, Italy) application. 

Postoperative antibiotic therapy was applied, with weekly tapering of steroids as prescribed treatment and artificial preservative-free tears. 

During the follow-up period, UCVA and BCVA were the same: one and three months postoperative 20/40, six months postoperative 20/30, and 36 months postoperative values were 20/22 (Table 1 (Tab. 1)). The same postoperative therapy was used after both protocols. 

As this is a case report, institutional review board approval was not applicable. 

## Discussion

The surgically treated eye with DISC protocol achieved better visual improvement for both UCVA and BCVA 36 months postoperatively, compared to the eye treated with Athens protocol where significant improvement was achieved in BCVA, while UCVA remained unchanged. A long-term study using the Athens protocol as the method for keratoconus treatment by Kanellopoulos et al. showed better improvement in postoperative BCVA (from 20/28 to 20/25) [10] and (from 20/34 to 20/25) [7] results compared to UCVA (from 20/39 to 20/31)10 and (from 20/100 to 20/36) [7] that converged with our report. 

Observing the central and maximal depth of laser ablation in both methods differences were noticed, being 13.86 µm in DISC protocol, and central ablation depth 28 µm and the maximum ablation depth 58 µm in the Athens protocol. This case showed that DISC protocol provided more advantageous results in corneal irregularity values where corneal irregularity decreased by 52% in the right eye and by 12.8% in the left eye. 

Conversely, values of spheric aberration in Zernike analysis 36 months postoperatively showed a lower increase in Athens protocol compared to DISC protocol. Unlike spheric aberration, Koma 0° and Koma 90° were similar in both procedures (Table 2 (Tab. 2)). 

Although keratometry values decreased compared to the preoperative value in both eyes, the decrease was greater in the right eye (Table 3 (Tab. 3)).

**Figure 2:**
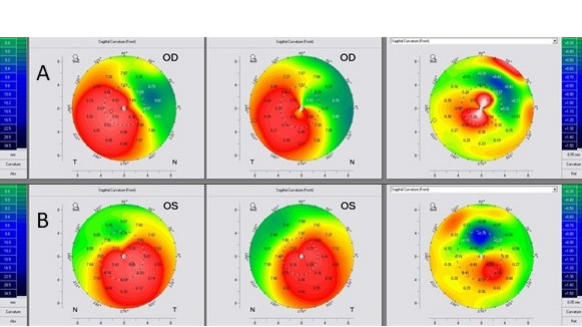
Comparison of preoperative and thirty-six months postoperative Oculyzer images of the right and left eye. A: right eye (DISC protocol), B: left eye (Athens protocol)

Kanellopoulos et al. showed a 15.31% decrease in Kmax in their study, which is twice larger than in our patient [10]. The analysis of topographic maps of the anterior corneal surface showed topographic regularization in both eyes, but more in the eye treated with Athens protocol as an expected outcome since regularization is considered one of the main outcomes of the procedure. However, the primary goal of the DISC protocol was not corneal regularisation, but rather an improvement of the patient’s refraction. Because laser ablation was performed in the pupil-apical line (the distance between the pupil center and corneal apex), some degree of corneal regularization was achieved (Figure 2 (Fig. 2)). In the Athens protocol, the main goal of TG-PRK is to minimize irregular astigmatism, make the corneal shape more regular and enhance BCVA [11], but not necessarily enhance UCVA, which may be the largest disadvantage for this procedure. In most cases, the laser treatment entails selective myopic and hyperopic ablations that can normalize the corneal surface and have a therapeutic impact. This effective surgical procedure helps to enhance BCVA and/or visual quality in several adverse situations [12].

**Figure 3:**
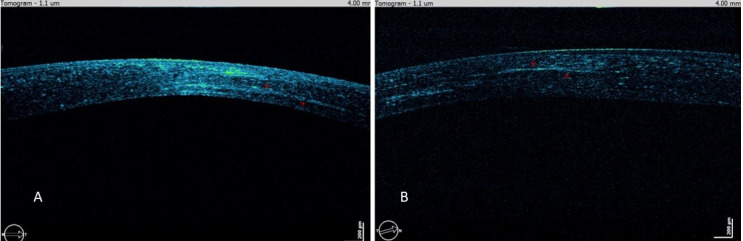
AOCT for the right and left eye 36 months postoperative. A: right eye (DISC protocol), B: left eye (Athens protocol)

Evaluating biomechanical stability and improvement in corneal strength after CXL, anterior segment optical coherence tomography (AOCT) was performed. A demarcation line (DL) was present in the corneal stroma which is an indirect sign that the tissue has hardened (Figure 3 (Fig. 3)) [13] in both eyes in different stromal depths.

Analysis of corneal indices showed lower values after two surgical procedures during the whole postoperative period (Table 4 (Tab. 4)). Index of height decentration (IHD, expressed in µm) measures vertical decentration of elevation data calculated using Fourier’s analysis on a ring with a radius of 3 mm and it had also been reducing throughout the entire postoperative period. We have noticed a greater decline of 66.9% in the value of IHD in the Athens protocol 36 months postoperative in comparison to the DISC protocol which correlates with the previous Athens protocol study of 53.3% decrease [10].

The reported 40 seconds of MMC usage is derived from senior author experience and stands within the range of reported data in the literature, where Coelho and Sieiro reported 30 seconds of use with 0.02 and 0.002% concentration with the same results in achieving corneal haze prevention [14], and Razmjoo et al. reported 45 seconds with 0.01 and 0.02%. In this case report, no corneal haze was noticed on either eye [15].

## Conclusion

The novel therapeutic approach presented in this paper, DISC protocol, did not show inferiority compared to the validated and widely used Athens protocol. Procedural optimization and a deeper comprehension of the underlying mechanisms require additional investigation and long-term follow-up that is currently under investigation. Possible advantages of this method are more superficial excimer laser depth ablation and more predictable visual outcomes. According to past clinical experiences and literature review, the DISC protocol offers good results and a high percentage of vision restoration in some patients, and thus has the potential to be a promising strategy for treatment of keratoconus, and visual ability improvement. 

## Notes

### Patient consent

The patient gave signed informed consent to be included in this case report.

### Acknowledgments and disclosures

All authors attest that they meet the current criteria of the International Committee of Medical Journal Editors Recommendations (ICMJE) for authorship.

**Other contributions:**

Lukrecija Levak, mag. med. techn., Knezović Eye Institute, Zagreb, Croatia

Danijel Marinić, opt. techn., Knezović Eye Institute, Zagreb, Croatia

### Data availability

Data are available with the permission of Knezović Eye Institute. The data that support the findings of this study are available from the corresponding author upon reasonable request.

### Competing interests

The authors declare that they have no competing interests.

## Figures and Tables

**Table 1:**
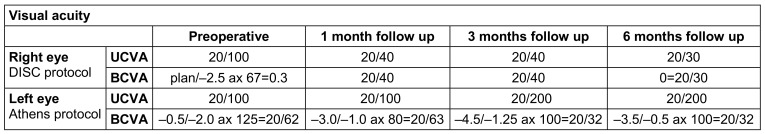
Visual acuity in both eyes pre- and postoperatively

**Table 2:**
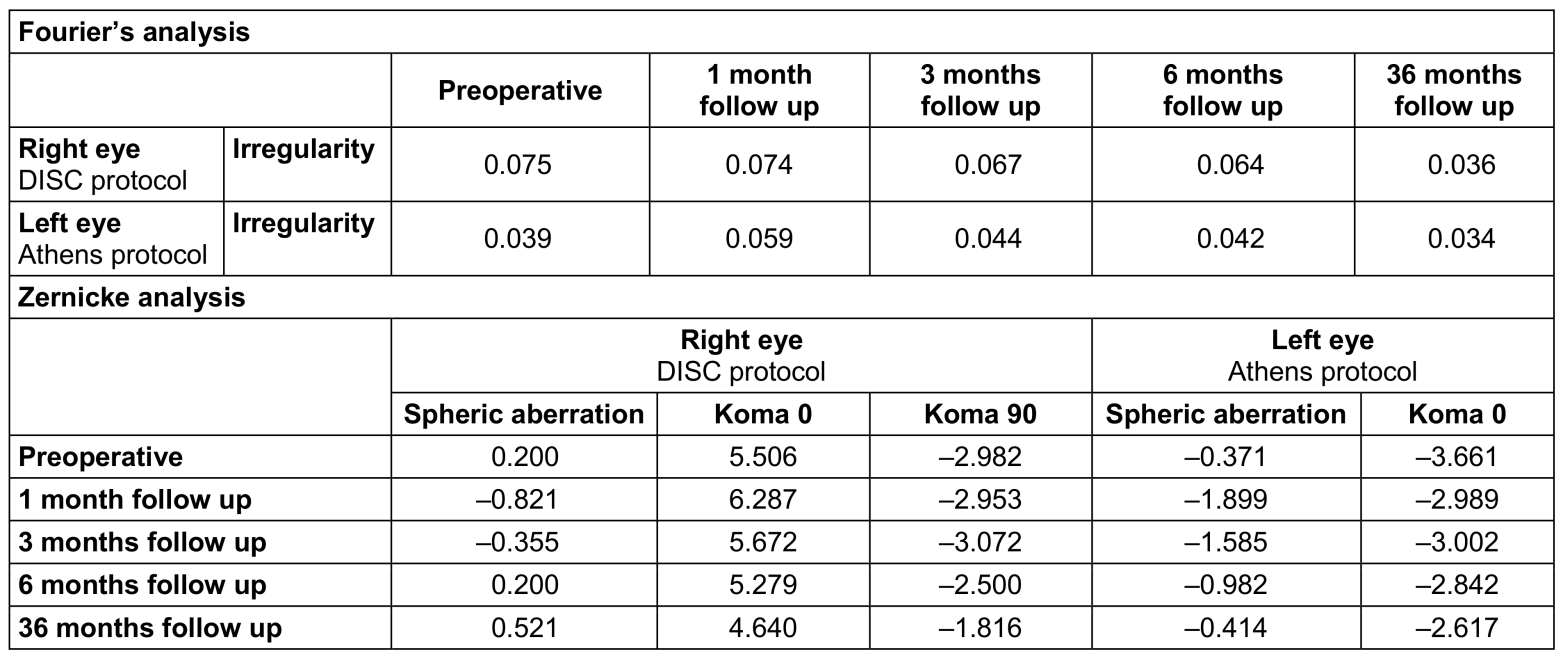
Fourier’s analysis and Zernike’s analysis

**Table 3:**
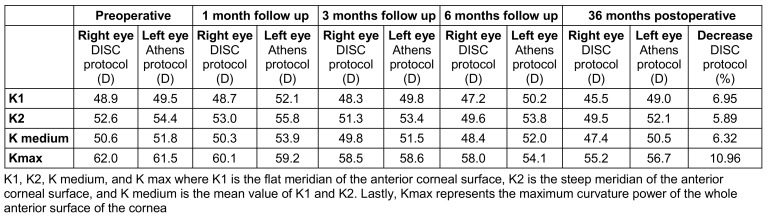
Keratometry values before surgery, one, 3, 6 and 36 months postoperative follow up

**Table 4:**
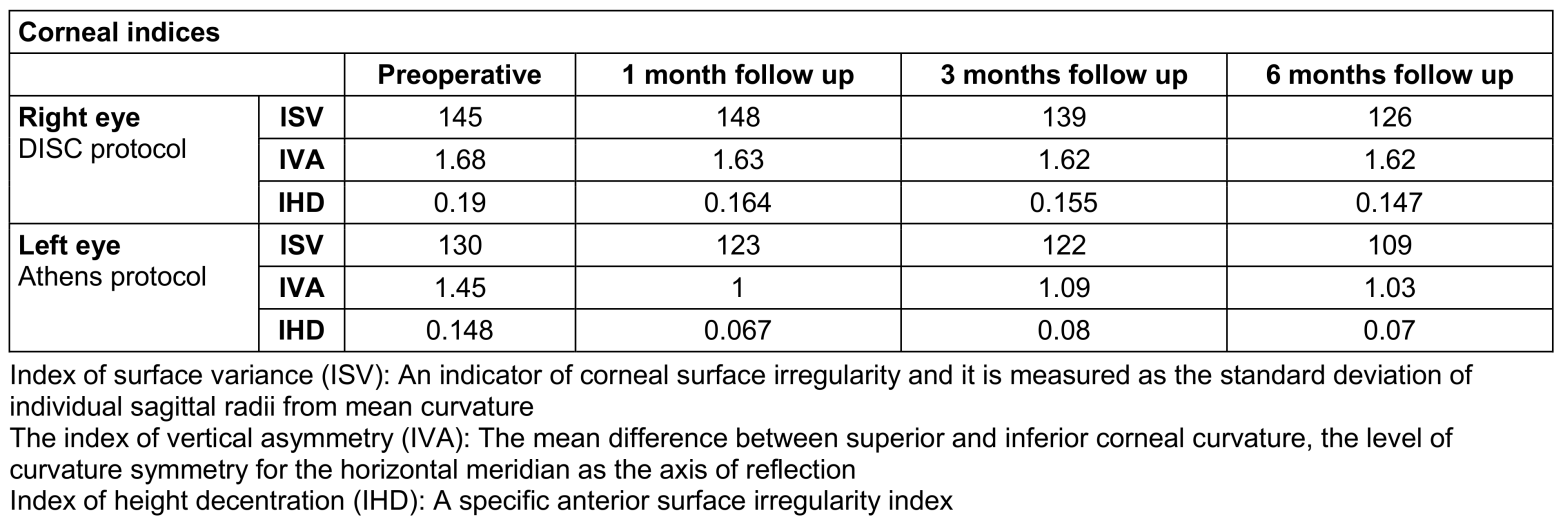
Values of corneal indices
